# Photoprotective Activity of *Buddleja cordata* Cell Culture Methanolic Extract on UVB-irradiated 3T3-Swiss Albino Fibroblasts

**DOI:** 10.3390/plants10020266

**Published:** 2021-01-30

**Authors:** Milton Abraham Gómez-Hernández, Miriam V. Flores-Merino, Jesús Enrique Sánchez-Flores, Cristina Burrola-Aguilar, Carmen Zepeda-Gómez, Aurelio Nieto-Trujillo, María Elena Estrada-Zúñiga

**Affiliations:** 1 Laboratorio de Toxicología de Productos Naturales, Escuela Nacional de Ciencias Biológicas, Instituto Politécnico Nacional (ENCB-IPN), Av. Wilfrido Massieu, Esq. Con Manuel M. Stampa s/n, Colonia Planetario Lindavista, Delegación Gustavo A. Madero, Ciudad de México C.P. 07700, Mexico; mgomezh1801@alumno.ipn.mx; 2 Facultad de Química, Universidad Autónoma del Estado de México, Paseo Colón s/n, Residencial Colón y Col Ciprés, Toluca C.P. 50120, Mexico; jsanchezflo@uaemex.mx; 3 Centro de Investigación en Recursos Bióticos-Facultad de Ciencias, Universidad Autónoma del Estado de México, Carretera Toluca-Ixtlahuaca Km 14.5, San Cayetano, Toluca C.P. 50295, Mexico; cba@uaemex.mx (C.B.-A.); anietot_ext@uaemex.mx (A.N.-T.); 4 Facultad de Ciencias, Universidad Autónoma del Estado de México, Campus El Cerrillo, Piedras Blancas, Carretera Toluca-Ixtlahuaca Km. 15.5, Toluca C.P. 50200, Mexico; zepedac@uaemex.mx

**Keywords:** *Buddleja cordata*, phenolic secondary metabolites, terpene secondary metabolites, UV radiation, photoprotection, cytotoxicity, in vitro culture, verbascoside

## Abstract

The research on compounds exhibiting photoprotection against ultraviolet radiation (UVR) is a matter of increasing interest. The methanolic extract of a cell culture of *Buddleja cordata* has potential photoprotective effects as these cells produce phenolic secondary metabolites (SMs). These metabolites are attributed with biological activities capable of counteracting the harmful effects caused by UVR on skin. In the present work, the methanolic extract (310–2500 µg/mL) of *B. cordata* cell culture showed a photoprotective effect on UVB-irradiated 3T3-Swiss albino fibroblasts with a significant increase in cell viability. The greatest photoprotective effect (75%) of the extract was observed at 2500 µg/mL, which was statistically comparable with that of 250 µg/mL verbascoside, used as positive control. In addition, concentrations of the extract higher than 2500 µg/mL resulted in decreased cell viability (≤83%) after 24 h of exposure. Phytochemical analysis of the extract allowed us to determine that it was characterized by high concentrations of total phenol and total phenolic acid contents (138 ± 4.7 mg gallic acid equivalents and 44.01 ± 1.33 mg verbascoside equivalents per gram of extract, respectively) as well as absorption of UV light (first and second bands peaking at 294 and 330 nm, respectively). Some phenylethanoid glycosides were identified from the extract.

## 1. Introduction

During recent decades, the search for new molecules showing photoprotection has increased as these can counteract the adverse effects provoked by ultraviolet radiation (UVR) [1,2,3,4,5]. UVR from sunlight is considered a carcinogenic agent and mainly promotes skin cancer [6,7,8]. This radiation is categorized into three types, regarding of wavelength, as UVC (100–280 nm), UVB (280–320 nm), and UVA (320–400 nm) [9,10]. The majority of UVR is absorbed by the ozone layer (100% of UVC, 90% of UVB, and a minimal amount of UVA); however, only UVA and UVB can pass through the atmosphere [11] and then penetrate the skin [12]. UVA can penetrate deep into the skin layer (epidermis and dermis), leading to DNA damage in human melanocytes and the overproduction of melanin and metalloproteinase as well as the production of reactive oxygen species (ROS), changes in inflammatory cells, and damage to fibroblast cells [5,13,14].

In epidermal cells, UVB is absorbed by chromophores such as aromatic amino acids, melanin, and nitrogenous nucleic acid bases; this triggers different cellular responses, such as apoptosis, melanogenesis, inflammation, ROS production, DNA repair mechanisms, and DNA mutations; the unrepaired mutations can progress to the development of skin cancer. In this way, acute or chronic exposure to UVA and UVB can cause several harmful effects to health, such as sunburn, edema, erythema, hyperpigmentation, photoaging, and skin cancer [10,12,15]. The use of photoprotection is highly recommended to reduce the damage caused by UVR on the skin [16]. The term photoprotection refers to protective factors that are categorized as being primary or secondary [17]. The former involves sunscreens that are physical or chemical barriers able to reflect, scatter, or absorb UV light [18,19,20,21].

However, certain compounds used in sunscreens can cause adverse effects, such as allergies or neurotoxicity [1,2]. The secondary factors are related to molecules possessing biological activities able to counteract the effects of UVR at the cellular and molecular level (e.g., anti-inflammatory, free radical scavenging, DNA repair, and anti-apoptotic activities). Therefore, research into new molecules showing photoprotection is a subject of increasing interest [3,5]. In this sense, phenolic compounds derived from plants might represent molecules exerting outstanding photoprotection as they exhibit intense UV absorption [22].

The chemical structure of phenolic compounds integrates one or more aromatic rings with hydroxyl group(s) as substituent(s) that show two bands in the UV absorption spectrum (around 280 and 300–360 nm) [23]. The hydroxyl substituent is able to donate hydrogen atoms to free radicals or to establish intermolecular interactions with the target molecules implicated in executing the adverse effects of UVR, such as cyclooxygenase-2 (COX-2), lipoxygenase, or nitric oxide synthase, which are enzymes involved in inflammatory processes [22]. The feasibility of evaluating the photoprotective potential of plants (and their contents) in UV-irradiated fibroblasts through measurements of cell viability and cytotoxicity using the 3-[4,5-dimethylthiazol-2-yl]-2,5-diphenyltetrazole bromide (MTT) assay has previously been demonstrated [24,25,26].

A methanolic extract of a cell culture of *Buddleja cordata* Kunth (Scrophulariaceae), a producer of verbascoside (at a high concentration of 116.36 mg/g dry weight biomass), linarin, and hydroxycinnamic acids (caffeic, ferulic, p-coumaric, and sinapic acids) [27] may represent a source of molecules with photoprotective effects. The *Buddleja* genus comprises 125 species distributed worldwide, with the majority of species (ca. 63) being reported in America; this continent has four centers of diversity, where México is outstanding in harboring 20% of this diversity [28,29,30].

Phytochemically, this genus synthesizes phenolic compounds (phenylethanoids and flavonoids) that are linked with conferring the medicinal properties exhibited by several species [29,31]. Such is the case of *B. scordioides* Kunth, whose extract is used as a sunscreen in Mexican folk medicine [32]; ethnopharmacological studies have demonstrated the photoprotective effect of this extract is due to the presence of verbascoside and linarin phenolic compounds that are responsible for this activity [33,34,35]. Verbascoside is a phenylethanoid glycoside recognized for its anti-inflammatory, in vitro DNA protective, and strong antioxidant effects [36,37]. Similarly, linarin is a flavonoid with antioxidant and anti-inflammatory activities [38].

Both secondary metabolites (SMs) have been identified in several *Buddleja* species distributed in Mexico, namely *B. americana L., B. cordata* Kunth, *B. parviflora* Kunth, and *B. scordioides* Kunth) [39]. It is well documented in the literature that SM production depends on genetic, biotic, and abiotic factors as well as the physiological conditions and development of the plant [40]; thus, the concentration in vegetal material might be inconstant, becoming an advantage when vegetal material might be used as source of bioactive SMs [41]. The in vitro culture of plants represents a biotechnological tool for the production of bioactive SMs as culture conditions can be manipulated to achieve high yields of production as well as to allow mass production using bioreactors [41].

For *B. cordata* Kunth, several biotechnological advances have been achieved with the aim of obtaining optimal culture conditions (e.g., elicitation with methyl jasmonate and bioreactor culture, among others) for high biomass and SM productions, especially concerning verbascoside [27,41,42,43]. This species has a wide territorial distribution in Mexico and is used by the Mexican folk population to counteract several diseases; its medicinal properties were found to be related to their constituent SMs [27]. Preclinical studies on acute and chronic inflammatory experimental models have demonstrated that a methanolic extract of *B. cordata* Kunth cell culture possessed anti-inflammatory effects that were better compared with the methanolic extract of leaves of the wild plant—an effect attributed to verbascoside and linarin [44,45]. The aim of the present work was to determine the photoprotective effect of the methanolic extract of *B. cordata* cell culture on UVB-irradiated 3T3-Swiss albino fibroblast cell line.

## 2. Results

### 2.1. Photoprotective and Cytotoxic Effects of the B. cordata Cell Culture Methanolic Extract on 3T3-Swiss albino Fibroblasts

**Figure 1:**
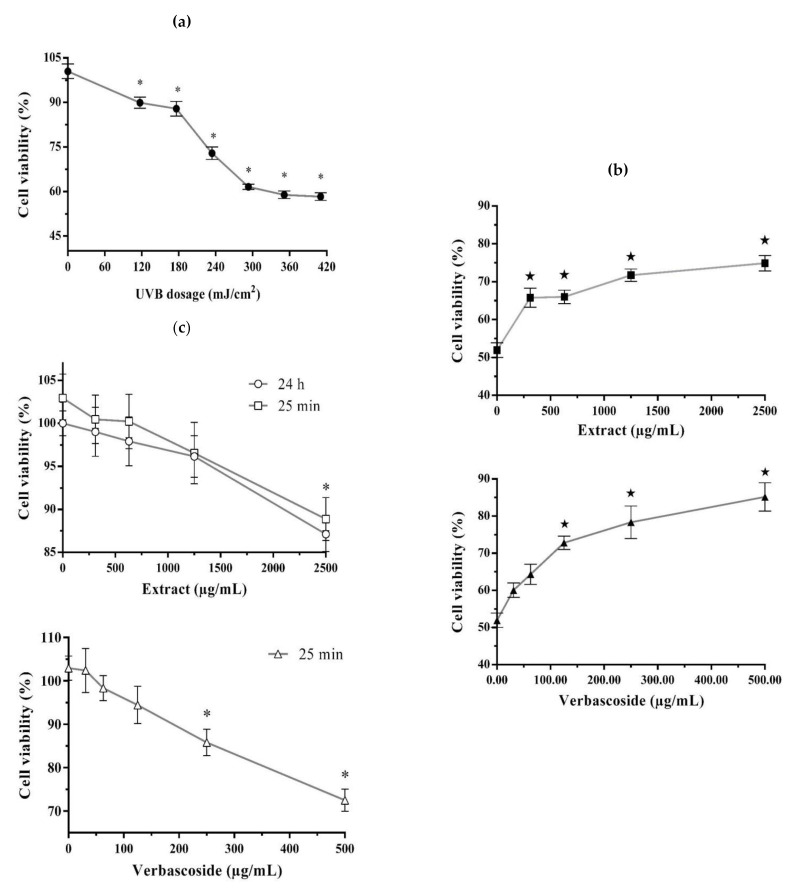
Photoprotective and cytotoxic effects of *B. cordata* cell culture methanolic extract on 3T3-Swiss albino fibroblasts: (**a**) effect of UVB irradiation energy dose (0–410 mJ/cm^2^) on the cell viability of fibroblasts (-●-); (**b**) photoprotective effect of the methanolic extract (0–2500 µg/mL) (-■-) and verbascoside (positive control; 0–500 µg/mL) (-▲-) in fibroblasts UVB-irradiated at 293 mJ/cm^2^; (**c**) cytotoxic effect of the methanolic extract (0–2500 µg/mL) after 25 min (-□-) and 24 h (-○-) of exposure or verbascoside (positive control; 0–500 µg/mL) after 25 min (-Δ-) of exposure. Data shown as average ± standard error of mean obtained from four experimental units in triplicate (*n* = 12). Within a tendency line, * indicates significant differences at the 5% significance level regarding treatment consisting of 0 µg/mL of the extract or verbascoside without UVB irradiation (0 mJ/cm^2^), while ^★^ indicates significant differences at the 5% significance level regarding treatment consisting of 0 µg/mL of the extract or verbascoside with UVB irradiation (293 mJ/cm^2^). In all experiments, a positive control for death was included using 23% (*v*/*v*) dimethylsulfoxide (DMSO), which resulted in cell viability of around 20% (data not shown).

The UVB radiation significantly decreased the cell viability of 3T3-Swiss albino fibroblasts in comparison with non-UVB-irradiated fibroblasts (100% cell viability). This effect depended on the tested UVR dose (117–410 mJ/cm^2^), with the lowest cell viability value (58–61%) reached under the 293, 351, and 410 mJ/cm^2^ doses (no statistically significant differences between them) (Figure 1a); the dose of 293 mJ/cm^2^ (25 min of exposure time and cell viability of 61%) was selected to conduct the subsequent photoprotection bioassay. The results of this bioassay showed that all tested concentrations of the *B. cordata* cell culture methanolic extract (310–2500 µg/mL) as well as verbascoside (positive control; 31–500 µg/mL) significantly increased the cell viability of UVB-irradiated fibroblast cells, and that this effect was concentration-dependent.

Hence, the best result in terms of increased cell viability occurred at extract concentrations higher than 1250 µg/mL (72–75% cell viability, with no statistically significant differences between them) and for verbascoside at concentrations higher than 250 µg/mL (78–85% cell viability, with no statistically significant differences between them) (Figure 1b). In addition, when non-UVB-irradiated 3T3-Swiss albino fibroblasts were exposed for 25 min and 24 h to the methanolic extract (310–2500 µg/mL), we observed that only the highest tested extract concentration significantly decreased the cell viability (89% and 87%, respectively) compared with non-UVB-irradiated fibroblasts without extract or verbascoside addition (100% cell viability) (Figure 1c). Concentrations of verbascoside higher than 250 µg/mL significantly decreased the cell viability (73–86%) of non-UVB-irradiated fibroblasts after 25 min of exposure (Figure 1c); thus, indicating a cytotoxic effect when the fibroblasts were exposed to either the extract or verbascoside at high tested concentrations.

**Figure 2:**
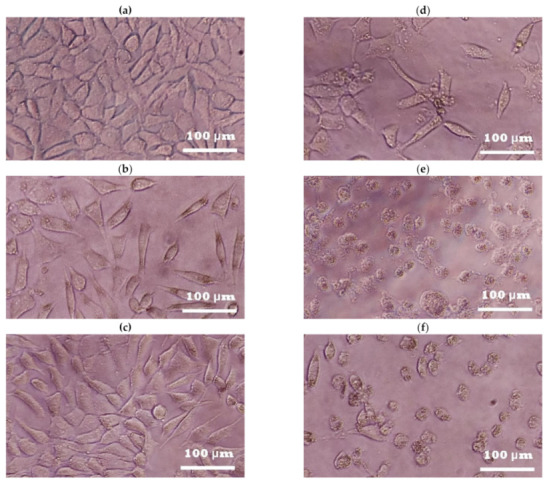
Changes in 3T3-Swiss albino fibroblast cell morphology during photoprotection and cytotoxicity assays: (**a**) without any treatment, (**b**) with extract (1250 µg/mL) and UVB irradiation (293 mJ/cm^2^, corresponding to 25 min of UVB exposure), (**c**) with extract (1250 µg/mL) at 25 min and 24 h exposure without UVB irradiation, (**d**) with UVB irradiation (293 mJ/cm^2^; corresponding to 25 min of UVB exposure), (**e**) non-UVB-irradiated cells supplied with cytotoxic concentrations of extract (2500 µg/mL; 25 min and 24 h of exposure), and (**f**) non-UVB-irradiated cells supplied with cytotoxic concentrations of verbascoside (>250 µg/mL; 25 min of exposure). The micrographs are from inverted microscopy using a 20× objective prior to measuring cell viability using the 3-[4,5-dimethylthiazol-2-yl]-2,5-diphenyltetrazole bromide (MTT) assay.

In general terms, the results of fibroblast cell viability in the photoprotection and cytotoxic bioassays were consistent with the observed typical spindle-shaped fibroblast morphology. The fibroblast cell culture without any treatment (Figure 2a; with control taken as 100% cell viability) covered a wide culture area and showed membrane projections and well-defined edges, commonly seen in healthy cells of this type [46], which was similar to that observed in the UVB-irradiated fibroblast cell culture with extract (Figure 2b) or with nontoxic concentrations of the extract and without UVB irradiation (Figure 2c). However, when the fibroblast cell culture was under UVB irradiation, the cells showed membrane retraction, size decrease, and irregular edges as well as low confluence (Figure 2d; control of UVB irradiation showing around 50% cell viability), which was also observed in the non-UVB-irradiated fibroblast cell culture supplied with cytotoxic concentrations of the extract (Figure 2e) or the verbascoside (Figure 2f).

### 2.2. The B. cordata Cell Culture Methanolic Extract Absorbs UV Light, Has a High Total Phenol Content, and Contains Bioactive SMs

**Figure 3:**
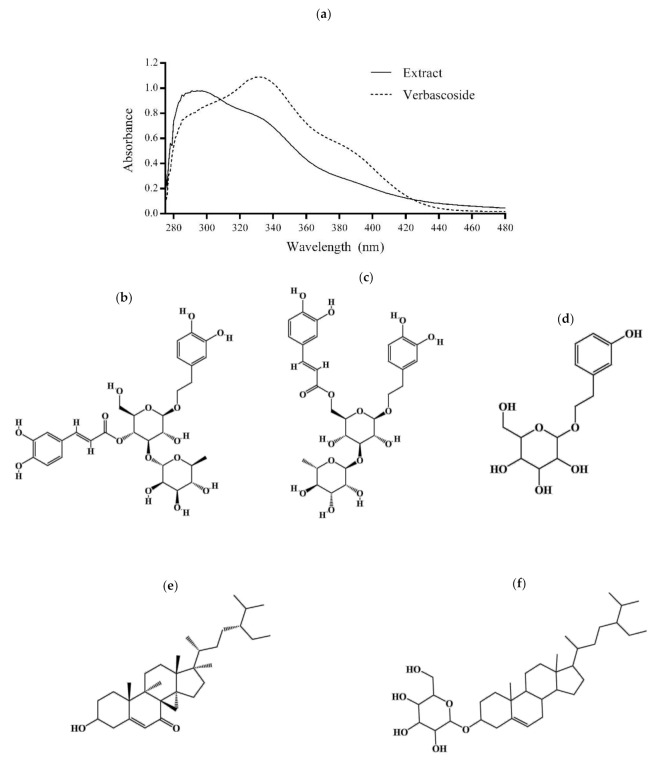
Phytochemical data of the *B. cordata* cell culture methanolic extract. (**a**) UV absorption spectrum (verbascoside was used as control for the extract) and secondary metabolites putatively identified by liquid chromatography mass spectrometry (LC/MS) in the extract: (**b**) verbascoside, (**c**) isoverbascoside, (**d**) 2-(3-hydroxyphenyl)ethanol 1′-glucoside, (**e**) 3-hydroxystigmast-5-en-7-one, (**f**) beta-sitosterol 3-O-beta-D-galactopyranoside.

The phytochemical analysis of the *B. cordata* cell culture methanolic extract showed an outstanding ability to absorb UV light from 280 to 400 nm (UVB to UVA) with the first band peaking at 294 (maximum) and a second band peaking at 330 nm (Figure 3a). This later peak was observed as the band peak corresponding to maximum absorption in the verbascoside spectrum (Figure 3a). Comparison of the absorption spectra (particularly at 330 nm) between the methanolic extract and verbascoside indicated a high concentration of verbascoside in the extract, whose presence was confirmed by putative identification of SMs through liquid chromatography mass spectrometry (LC/MS) (Figure 3b).

The LC/MS analysis also allowed us to identify putatively certain SM-type phenolics (phenylethanoid glycosides) such as isoverbascoside (Figure 3c) and 2-(3-hydroxyphenyl)ethanol 1′-glucoside (Figure 3d) as well as 3-hydroxystigmast-5-en-7-one (Figure 3e) and beta-sitosterol 3-O-beta-D-galactopyranoside (Figure 3f) terpene SMs. The methanolic extract had a total phenol content of 138 ± 4.7 mg gallic acid equivalents per gram of extract and a total phenolic acid content of 44.01 ± 1.33 mg verbascoside equivalents per gram of extract.

## 3. Discussion

**Figure 4:**
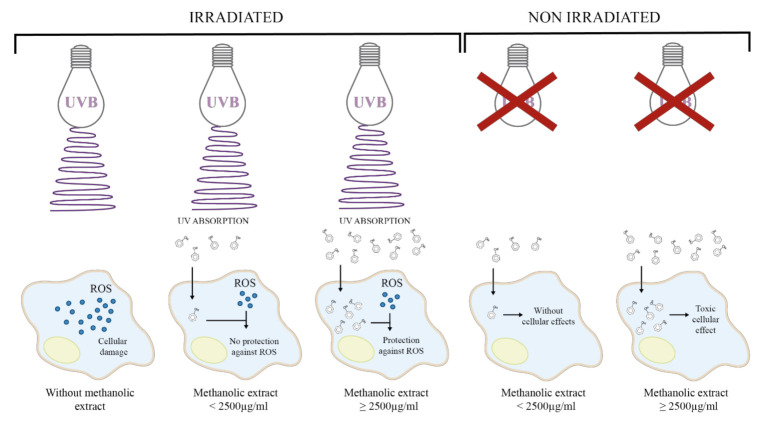
Proposed molecular characteristics of the *B. cordata* cell culture methanolic extract that underlie its photoprotective activity and cytotoxic effects on 3T3-Swiss albino fibroblasts.

The energy from UV irradiation decreased L292 fibroblast cell viability in a dose-dependent manner (100 to 800 mJ/cm^2^) [24,25], and in particular, a dose of 600 mJ/cm^2^ decreased the cell viability by around 50% [24]. In this work, a UVB energy dose of 293 mJ/cm^2^ reduced the cell viability of 3T3-Swiss albino fibroblasts by around 60% (Figure 1a). Fibroblasts are cells of the dermis that are responsible for producing outstanding components of the extracellular matrix (e.g., elastin, collagen, and hyaluronic acid) that confer elasticity and strength to the skin. UVR causes degradation of the dermis fibers, leading to photoaging [12,47]. Additionally, it has been reported that a decrease in cell viability under UVB irradiation is the result of apoptosis as it is induced by the inability of cells to repair DNA damage caused by ROS [48]. UVB energy increases the transcription of pro-apoptotic genes or those involving cell cycle arrest [49,50]. In our study, we observed that the methanolic extract of *B. cordata* cell culture provided photoprotection to UVB-irradiated 3T3-Swiss albino fibroblasts since the results showed an increase in cell viability with regard to cells that were solely UVB-irradiated (Figure 1b). This photoprotective effect (Figure 4) can mainly be explained by the ability of the extract to absorb UV light (Figure 3a), an ability that phenolic compounds possess due to their structure [23]; thus, the photoprotective effect of the extract can be attributed to the phenylethanoid glycosides putatively identified by LC/MS (Figure 3b–d). According to the results, phenolics were found in the methanolic extract at high concentrations, where verbascoside could be one of the major components of the extract [27,42,43], thus contributing to the fact that the methanolic extract at 1250 µg/mL had the same effect on cell viability (72%) of UVB-irradiated fibroblasts as verbascoside at 125 µg/mL (73%) (Figure 1b). However, the maximum peak of the extract at 294 nm, observed in the absorption spectra, indicates that the extract contains other phenolic compounds, which might be cinnamic acid derivatives, such as caffeic and ferulic acids that are known to be produced by *B. cordata* cells [27], and whose maximum peak has been reported near 290 nm [51]. Future research should be conducted to isolate the SMs responsible for the photoprotective effects in the methanolic extracts of *B. cordata* cell culture, since the LC/MS data not only showed the presence of phenylethanoid glycosides (Figure 3b–d) but also sterols (Figure 3e,f), and other important bioactive SMs reported in *Buddleja* species could be present in the extract, such as phenolic compounds (flavonoid and phenolic acid) and terpenes (essential oils, iridoid, saponin, sesquiterpene, and triterpene) [39]. In addition, new investigations regarding the importance of phenolic compound production in the physiology and ecology of *B. cordata* should be performed in future studies as the synthesis of phenolic compounds is considered an evolutionary mechanism by which plants mitigate the harmful effects of UVR through the scavenging of free radicals [52]. Due to their antioxidant properties, these compounds could be involved in the different defense responses of plants to counteract ROS production triggered by biotic and abiotic stresses [53]. Verbascoside absorption has been reported to occur rapidly (between 5 and 15 min in human colonic tissues, reaching maximum tissue concentrations between 15 and 30 min [54]. While, after 13 min of oral administration in rats, verbascoside reached a plasmatic concentration of 135 ng/mL [55]), which highlights the probability of uptake of the methanolic extract of *B. cordata* cell culture by the 3T3-Swiss albino fibroblasts. However, it appears that concentrations of the extract >1250 µg/mL resulted in major amounts of extract being uptaken inside cells compared with lower concentrations (<1250 µg/mL), where the high amounts extract that were uptaken resulted in the best photoprotective effect as observed in photoprotection bioassays (Figure 1b). While the extract at 2500 µg/mL, whether after 25 min or 24 h of exposure, was shown to be toxic in the cytotoxic bioassay (Figure 1c) (Figure 4). Thus, the best photoprotective effect of the methanolic extract was likely not only due to the absorption of UVB light by phenolic compounds but also by stimulation of cellular protection, which resulted in counteracting ROS damage (antioxidant effect) (Figure 4). In general terms, phenolic compounds are antioxidant agents [22,56], although verbascoside is recognized as being particularly potent [36] with an 50% inhibitory concentration (IC_50_) for 2,2-diphenyl-1-picrylhydrazyl (DPPH) radicals of 1.56 µg/mL. This is better than that reported for ascorbic acid (used as a common reference standard), which is 74.32 µg/mL [57], while the IC_50_ under 2,2′-azino-bis-[3-ethylbenzothiazoline-6-sulfonic acid] diammonium salt (ABTS) radicals is 5.36 µg/mL [58]. Verbascoside acted as an in vitro DNA protector at 312 µg/mL [59], and this effect is related to oxidative stress reduction. Isoverbascoside, also putatively detected in the methanolic extract (Figure 3c), has also been reported to be an antioxidant [60]. However, at concentrations of the extract lower than 1250 µg/mL, it is suggested that the levels of phenolic compounds inside the fibroblasts were low; thus, they were not able to counteract ROS damage and were mainly reliant on UV absorption for photoprotective effects (Figure 4).

Likewise, the biological effects of the *B. cordata* cell culture methanolic extract at the highest tested concentration on fibroblasts were comparable to verbascoside (Figure 1b,c), thus, indicating the importance of the concentration in exerting significant biological effects. In other studies, the exposure of V79 fibroblasts and HaCaT keratinocytes to verbascoside over 24 h significantly reduced the cell viability by around 50% (78.8 and 31.23 µg/mL, respectively) [61,62]. This suggests that the cytotoxic effect of the verbascoside is caused by its ability to reduce the activity of cytoplasmic protein tyrosine phosphatases by binding to the Scr homology 2 (SH2) NH2-terminal domain (SHP1); these receptors are implicated in the growth, differentiation, and apoptosis of cells [37,63,64]. The application of verbascoside at 644 µg/mL avoided around 65% of the necrosis induced by UVC radiation in keratinocytes [65,66]. The concentration of 62.4 µg/mL for verbascoside inhibited the expression of nuclear factor-κB in a THP-1 cell line [67], while the concentration of 31.2 µg/mL inhibited COX-2 activity in the U937 cell line [37], resulting in decreased inflammation in both cases. In a murine acute inflammation model under UV irradiation, the photoprotection effect of verbascoside (2000 µg/mL) was validated through the reduction of erythema of around 45% [34]. Verbascoside (2.5–25 µg/mL) showed significant wound healing effect in HaCaT cell line in vitro and Wistar rats in vivo models [68], which might contribute to counteracting the harmful effects of UVR. It also acted as a lipid protector by decreasing lipoperoxidation at a dose of 800 mg/kg in an in vivo rabbit biological model [69]. In addition, verbascoside has shown other biological effects related to photoprotection, such as anti-irritant, melanin inhibitor, cytoprotective, analgesic, chemopreventive, and chemotherapeutic effects [36]. In pre-clinical studies, *B. cordata* cell culture methanolic extract, containing verbascoside, demonstrated its potential as an anti-inflammatory, and this effect was significantly higher than that shown by the methanolic extract of wild *B. cordata* plant leaves. The cell extract at 2 mg/ear caused inhibition of the edema by 61.72%, while in an acute model, 200 mg/kg caused inhibition of subplanar edema by 49%; in both cases, the effects were not considered statistically different to those of the indomethacin reference drug. The cell extracts at 2 g/kg did not show toxicity in an acute mice model [44]. In the chronic inflammation murine model, cell extracts at 250 mg/kg on day 28 reduced edema by 67% as well as reduced oxidative stress and the levels of interleukin-1β, tumor necrosis factor-α, and CD4+ lymphocytes in ganglionic tissue and increased the levels of the anti-inflammatory cytokine interleukin-10. Additionally, at 1 g/kg, the *B. cordata* cell culture methanolic extract was not lethal or toxic after 28 days of administration [45]. These data clearly show the necessity to control the dose of the bioactive SM, and it is possible that the poor photoprotective effect of the methanolic extract of a cell culture of *B. cordata* at the lower tested concentrations was caused by low bioavailability of the bioactive SMs. Phytochemicals from plants, such as phenolic compounds, have shown low bioavailability [70,71], and this includes verbascoside, whose cellular concentration in human colonic tissues was 267 pmol/mg of cellular protein, representing a total accumulation efficiency of approximately 0.12% with an accumulation efficiency of 0.1% [54]. Finally, there are several works reporting the improvement of bioavailability and stability of verbascoside under liposomal encapsulation [72,73,74,75]. Thus, since the *B. cordata* cell culture methanolic extract was shown to have a high concentration of phenolic compounds, future research must focus on improving its pharmacokinetics in addition to carrying out future in vivo studies related to chronic UVB radiation exposure. Among the harmful effects of UVA and UVB, the latter has been reported as a more genotoxic agent since it drives skin carcinogenesis [76]. UVA has been related to hyperpigmentation and photoaging [5,13], while UVB provokes sunburns and skin cancer [12]. It has been demonstrated that skin cancer is not exclusive to Caucasian populations [77] but can also be developed in other populations, such as Mexican, where a high prevalence of basal cell skin cancer is reported [78,79,80].

## 4. Materials and Methods

### 4.1. Preparation of the Methanolic Extract and Verbascoside Standard

The dry methanolic extract of *B. cordata* Kunth (Scrophulariaceae) cell culture [27] was provided by the Centro de Investigación en Recursos Bióticos of the Universidad Autónoma del Estado de México (UAEMex). To determine the photoprotective or cytotoxic activity, an extract stock solution (50 mg/mL) was prepared, which was then diluted to obtain different concentrations (3.1, 6.3, 12.5, and 25.0 mg/mL). A stock solution (5 mg/mL) of verbascoside (Sigma-Aldrich, Saint Louis, MO, USA), used as a positive control, was separately prepared and diluted to obtain different concentrations (0.31, 0.63, 1.25, and 2.5 mg/mL).

The tested concentrations used in bioassays were defined according to previous works regarding the photoprotection of verbascoside [33,34,35], as well as its cytotoxicity [62], and its high concentration in *B. cordata* Kunth cell culture (116.36 mg of verbascoside/g dry weight biomass and 152 mg of verbascoside/g dry weight extract) [27,44]. These solutions were used in subsequent assays to obtain different concentrations for the extract and verbascoside.

The solutions tested (stock and dilutions) for the photoprotective activity were prepared in Dulbecco’s modified Eagle’s medium (without glucose, L-glutamine, phenol red, sodium pyruvate, and sodium bicarbonate) (Sigma-Aldrich, Saint Louis, MO, USA), hereinafter identified as DMEM, while those solutions tested (stock and dilutions) for cytotoxicity were prepared in high glucose Dulbecco’s modified Eagle’s medium (with 4500 mg/L glucose, L-glutamine, and sodium pyruvate, without sodium bicarbonate; Sigma-Aldrich, Saint Louis, MO, USA) supplemented with 5% (*v/v*) fetal bovine serum (FBS; Biowest, Nuaillé, Pays De La Loire, Francia), 100 U/mL of penicillin, and 100 µg/mL of streptomycin (Sigma-Aldrich, Saint Louis, MO, USA), hereinafter identified as DMEM-HG.

### 4.2. In Vitro Bioassays

#### 4.2.1. Conditions of the Cell Culture

The 3T3-Swiss albino mouse fibroblast cell line (ATCC^®^CCL-92^TM^) was used in the present work. The cells were cultured with DMEM-HG and incubated at 37 °C, under a 5% CO_2_ atmosphere and 85% humidity. Prior to the in vitro bioassays, fibroblast cells were cultured for 24 h in 96-well plates at a density of 8 × 10^3^ cells per well. The medium was removed after 24 h of incubation.

#### 4.2.2. Determination of the Photoprotective Activity of the Extract

##### Irradiation and Selection of the UVB Energy Dose

Prior to the photoprotection bioassay, the effect of UVB energy doses on 3T3-Swiss albino fibroblast cell viability was determined. UVB irradiation was carried out using a UVB150 Exo Terra Reptile 25W lamp (Figure S7) with an intensity of 195 mW/cm^2^ (China) and was placed 10 cm over the microplate. The doses of UVB energy tested (0–410 mJ/cm^2^) were determined according to Antonio-Gutiérrez et al. [81], as shown in the following equation:

(1) $$\mathrm{Dose}\;\left(\frac{\mathrm{mJ}}{\mathrm{cm}2}\right)=\;\mathrm{Intensity}\;\left(\frac{\mathrm{mW}}{\mathrm{cm}2}\right)\text{×}\mathrm{Exposure}\;\mathrm{time}\left(s\right)$$

The culture medium was removed prior to UVB irradiation, and 100 µL of fresh DMEM was then added per well; this culture medium was selected to avoid possible interference due to the phenol group from phenol red and phenolic compounds of the extract or verbascoside. Then, the cells were irradiated for 0, 10, 15, 20, 25, 30, or 35 min for the different UVB doses. At the end of the assay, the medium in each well was replaced with 100 µL of DMEM-HG. After 24 h of incubation, the percentage of viable cells relative to non-irradiated cells was estimated by MTT cell assay, which measures the metabolic activity of live cells and is used as an indicator of cell viability. The UVB energy dose that provoked around 50% cell inhibition was selected for the photoprotection bioassay.

##### Photoprotective Bioassay

For the photoprotective bioassay, the medium in the cell cultures was removed after 24 h incubation, then 90 µL of fresh DMEM and 10 µL of the different solutions of the extract or the verbascoside (described in Section 4.1) were added per well. The final concentrations of the extract were 310, 630, 1250, and 2500 µg/mL and were 31, 63, 125, 250, and 500 µg/mL for verbascoside. The control consisted of 10 µL DMEM without extract or verbascoside. After 10 min of sample addition (extract or verbascoside), the cells were irradiated at the previously selected UVB dose (293 mJ/cm^2^ equal to 25 min of UVB exposure). At the end of the bioassay, the medium in each well was replaced with 100 µL of DMEM-HG. Subsequently, after 24 h of incubation, micrographs were taken using inverted microscopy and a 20× objective. The cell viability was then measured by MTT assay.

#### 4.2.3. Determination of the Cytotoxicity of the Extract

A simultaneous bioassay was run under the same conditions described for the photoprotection test but without UVB irradiation in order to determine the effect of the extract and verbascoside after 25 min of exposure. In addition, the effect of the extract after 24 h of exposure was observed and analyzed; thus, after 24 h incubation of the fibroblast cells, the medium was replaced with 90 µL of fresh DMEM-HG and 10 µL of the different solutions of extract (described in Section 4.1) to obtain different concentrations per well (0, 310, 630, 1250, and 2500 µg/mL). Subsequently, the cells were incubated for 24 h and micrographs were captured using inverted microscopy and a 20× objective. Cell viability was then determined using MTT assay.

#### 4.2.4. Determination of Cell Viability

The MTT assay was used to determine cell viability; it is based on the reduction of MTT to formazan by the mitochondrial dehydrogenase of metabolically active cells. It establishes a direct relationship between the amount of formazan generated and the metabolic activity of live cells [26]. The procedure consists of removing the culture medium of every well and replacing it with 100 µL of fresh DMEM and 50 µL of MTT (1.5 mg/mL; prepared in Dulbecco’s phosphate-buffered saline; ATCC, USA). Then, the samples were incubated in the dark for 3 h (5% CO_2_, 85% humidity, 37 °C). After incubation, the supernatant of the wells was replaced with 100 µL DMSO. The cell culture well plates were placed in an orbital shaker (Orbit P2, Labnet, Edison, NJ, USA) for 10 min. Finally, the supernatants of the wells were transferred onto a new microplate, and the absorbance was recorded at 570 nm using a microplate reader (Multiskan™ FC Microplate Photometer, Thermo Scientific, Waltham, MA, USA). The percentage of viable cells was estimated using the following equation.

(2) $$\mathrm{cell}\;\mathrm{viability}\;\left(\%\right)=\frac{\mathrm{sample}\;\mathrm{absorbance}}{\mathrm{control}\;\mathrm{absorbance}\;}\times 100$$

where the control absorbance was cells without any treatment, i.e., untreated cells.

In each of the previously described bioassays, an internal death control was simultaneously carried out under the same conditions as the samples. For the death control, 100 µL of DMEM-HG at 23% (*v/v*) DMSO was added at the end of the assay; the cell viability registered under the MTT assay for this control was 20%.

### 4.3. Phytochemical Analysis

#### 4.3.1. Measurement of the UV Absorption Spectrum

A sample (50 µL) of the methanolic extract (2.5 mg/mL) or verbascoside (0.25 mg/mL) was diluted in distilled water to a final volume of 3 mL. These dilutions were separately analyzed to obtain the absorption spectrum at a range from 280 to 480 nm in a UV–visible spectrometer (Evolution 60S, Thermo Scientific, Waltham, MA, USA). The corresponding blank control was prepared with DMEM.

#### 4.3.2. Quantitative Analysis

The total phenol and total phenolic acid contents of the methanolic extract (2 mg/mL) were determined using a procedure described by Vazquez-Marquez et al. [41]. Gallic acid (Sigma-Aldrich, USA) was used to build a calibration curve (0.023–0.375 mg/mL; y = 2.6609 x − 0.019, R^2^ = 0.997285) for the total phenol content and the obtained results were expressed as mg gallic acid equivalents per gram of extract. Verbascoside (Sigma-Aldrich, Saint Louis, MO, USA) was used to build a calibration curve (0.16–2.5 mg/mL; y = 0.7508 x + 0.0449, R^2^ = 0.9985) for the total phenolic acid content, and the corresponding results were expressed as mg verbascoside equivalents per gram of extract.

#### 4.3.3. Identification of Extract Secondary Metabolites through LC/MS

A sample (10 µL) of the methanolic extract (0.5 mg/mL) was analyzed by LC/MS to identify the SMs. Separation was carried out using an LC/MS system (UHPLC 1290 Infinity II/Q-TOF 6545 Agilent Technologies, Santa Clara, CA, USA) consisting of a G7104A Quat pump, G7129B sampler, and G7130A column oven (Agilent Technologies, Santa Clara, CA, USA) connected to a Q-TOF G6545A mass spectrometer. The column oven was set at 40 °C. The elution was performed on a column ACQUITY UPLC BEH C18 of 1.7 µm, 2.1 mm × 50 mm. The mobile phase consisted of (A) water and (B) acetonitrile with 0.1% formic acid, which was applied in the following gradient elution: 98% in first 2 min A, 2–25 min 98–30% A, 25–28 min 30% A, 28–30 min, 30–5%, 30–32 min 5% A, 32–35 min 5–98% A, and 35–37 min 98% A. The flow rate was set to 0.4 mL/min. The column was thermostated at 40 °C. The injection volume was 10 µL. Three replicates were analyzed for all samples.

A Q-TOF LC/MS G6545A mass spectrometer equipped with a dual AJS ESI ion source was used with gas temp of 300 °C, drying gas of 7 L/min, nebulizer 25 psig, sheath gas temp of 300 °C, sheath gas flow of 8 L/min, and skimmer offset 65 V with a fragmentor voltage of 120 V. The MS data were acquired in negative and positive mode, with a mass range from 100 to 3000 *m/z*. Multiple mass spectrophotometric scanning modes, including full scanning, product ion scanning (PIS), and negative and positive scanning were conducted for the qualitative analysis. Collision-induced fragmentation experiments were performed using nitrogen as the collision gas and with the collision energy set to 5, 10, and 15 V. A selection reaction monitoring experiment for quantitative analysis was performed using two MS fragments for each compound which were previously defined as dominant in PIS experiments. The SMs were identified by direct comparison with Metlin_Metabolites_AM_PCD.cdb database. The corresponding results were recovered (Figures S1–S6).

### 4.4. Statistical Analysis

All treatments consisted of four experimental units in triplicate (*n* = 12). All data were verified for homoscedasticity and normality. The values for cell viability obtained from photoprotection and cytotoxicity bioassays were analyzed using one-way ANOVA followed by the Tukey–Kramer post-hoc test for multiple comparison. Sigma Plot software (version 12) was used for all statistical analyses. A *p*-value less than 0.05 was considered to indicate significant differences in all statistical analyses.

## 5. Conclusions

The methanolic extract of *Buddleja cordata* cell culture exerted a photoprotective effect on UVB-irradiated 3T3-Swiss albino mouse fibroblast cell line, which was due to the absorption of UV light by phenolic compounds present in the extract, such as phenylethanoid glycosides putatively identified by LC/MS. Future research must focus on the methanolic extracts of *B. cordata* cell culture in order to isolate the SMs responsible for the photoprotective effects, carry out future in vivo studies related to chronic UVB radiation exposure, and improve its pharmacokinetics.

## Data Availability

The data presented in this study are available within the article or Supplementary Material.

## Supplementary Materials

The following are available online at https://www.mdpi.com/2223-7747/10/2/266/s1. Figure S1: LC/MS chromatogram in electrospray (**a**) negative-ion and (**b**) positive-ion mode of the methanolic extract of *Buddleja cordata* cell culture, where 2-(3-hydroxyphenyl)ethanol 1′-glucoside, verbascoside, isoverbascoside, beta-sitosterol 3-O-beta-D-galactopyranoside, and 3-hydroxystigmast-5-en-7-one were detected at 1.959, 7.162, 7.177, 24.439, and 26.261 min, respectively. Figure S2: Mass spectrum of verbascoside (C_29_H_36_O_15_) in electrospray negative-ion mode detected in the methanolic extract of *Buddleja cordata* cell culture. Figure S3: Mass spectrum of isoverbascoside (C_29_H_36_O_15_) in electrospray negative-ion mode detected in the methanolic extract of *Buddleja cordata* cell culture. Figure S4: Mass spectrum of 2-(3-hydroxyphenyl)ethanol 1′-glucoside (C_14_H_20_O_7_) in electrospray negative-ion mode detected in methanolic extract of *Buddleja cordata* cell culture. Figure S5: Mass spectrum of 3-hydroxystigmast-5-en-7-one (C_29_H_48_O_2_) in electrospray positive-ion mode detected in the methanolic extract of *Buddleja cordata* cell culture. Figure S6: Mass spectrum of beta-sitosterol 3-O-beta-D-galactopyranoside (C_35_H_60_O_6_) in electrospray negative-ion mode detected in the methanolic extract of *Buddleja cordata* cell culture. Figure S7: UV–visible spectrum of UVB150 Exo Terra Reptile 26W lamp used for UVB irradiation of 3T3-Swiss albino fibroblasts. The UVB150 Exo Terra Reptile has a high UVB output similar to that of sunlight in deserts (http://www.exo-terra.com/en/products/reptile_uvb150.php).

## References

[1] Scheuer E., Warshaw E. (2006). Sunscreen Allergy: A Review of epidemiology, clinical characteristics, and responsible allergens. Dermatitis.

[2] Ruszkiewicz J.A., Pinkas A., Ferrer B., Peres T.V., Tsatsakis A., Aschner M. (2017). Neurotoxic effect of active ingredients in sunscreen products, a contemporary review. Toxicol. Rep..

[3] Giraldo J.C., Atehortúa L., Mejía M.A. (2014). Foto-protección: Mecanismos bioquímicos, punto de partida hacia mejores filtros solares. Dermatología Cosmética Médica Quirúrgica.

[4] Gilaberte Y., González S. (2010). Novedades en fotoprotección. Actas Dermo-Sifiliogr..

[5] Latha M.S., Martis J., Shobha V., Shinde R.S., Bangera S., Krishnankutty B., Bellary S., Varughese S., Rao P., Kumar B.R.N. (2013). Sunscreening agents: A review. J. Clin. Aesthet. Dermatol..

[6] Lee T., Sigurdson A.J., Preston D.L., Cachoon E.K., Freedman D.M., Simon L.S., Nelson K., Matanoski G., Kitahara C.M., Liu J.J. (2015). Occupational ionizing radiation and risk of basal cell carcinoma in US radiologic technologists (1983–2005). Occup. Environ. Med..

[7] Xiang F., Lucas R., Hales S., Neale R. (2014). Incidence of Nonmelanoma skin cancer in relation to ambient UV radiation in white populations, 1978–2012 Empirical Relationships. JAMA Dermatol..

[8] Moan J., Grigalavicius M., Baturaite Z., Dahlback A., Juzeniene A. (2014). The relationship between UV exposure and incidence of skin cancer. Photodermatol. Photoimmunol. Photomed..

[9] González-Púmariega M., Tamayo M.V., Sánchez-Lanar A. (2009). La radiación ultravioleta su efecto dañino y consecuencias para la salud humana. Theoria.

[10] Ravanat J.L., Douki T. (2016). UV and ionizing radiations induced DNA damage, differences and similarities. Radiat. Phys. Chem..

[11] Holick M.F. (2016). Biological effects of sunlight, ultraviolet radiation, visible light, infrared radiation and vitamin D for health. Anticancer Res..

[12] Young A.R., Claveau J., Rossi A.B. (2017). Ultraviolet radiation and the skin: Photobiology and sunscreen photoprotection. J. Am. Acad. Dermatolo..

[13] Panich U., Tangsupa-a-nan V., Onkoksoong T., Kongtaphan K., Kasetsinsombat K., Akarasereenont P., Wongkajornslip A. (2011). Inhibition of UVA-mediated melanogenesis by ascorbic acid through modulation of antioxidant defense and nitric oxide system. Arch. Pharm. Res..

[14] Ortonne J.P. (2002). Photoprotective properties of skin melanin. Br. J. Dermatolo..

[15] D’Orazio J., Jarrett S., Amaro-Ortiz A., Scott T. (2013). UV Radiation and the skin. Int. J. Mol. Sci..

[16] American Academy of Dermatology. https://www.aad.org/public/spot-skin-cancer/learn-about-skin-cancer/prevent.

[17] Rai R., Shanmuga S.C., Srinivas C.R. (2012). Update on photoprotection. Indian J. Dermatol..

[18] Skin Cancer Foundation. https://www.skincancer.org/prevention/sun-protection/sunscreen.

[19] Ulrich C., Jürgensen J.S., Degen A., Hackethal M., Ulrich M., Patel M.J., Eberle J., Terhorst D., Sterry W., Stcokfleth E. (2009). Prevention of non-melanoma skin cancer in organ transplant patients by regular use of a sunscreen: A 24 months, prospective, case-control study. Br. J. Dermatol..

[20] Ghiasvand R., Weiderpass E., Green A.C., Lund E., Veirod B. (2016). Sunscreen use and subsequent melanoma risk: A population-based cohort study. J. Clin. Oncol..

[21] Green A.C., Williams M.G., Logan V., Strutton G.M. (2011). Reduced melanoma after regular sunscreen use: Randomized trial follow-up. J. Clin. Oncol..

[22] Belščak-Cvitanović A., Durgo K., Hudek A., Bačun-Družina V., Komes D., Ganalakis C.M. (2018). Overview of polyphenols and their properties. Polyphenols: Properties, Recovery, and Applications.

[23] Solovchenko A.E., Merzlyak M.N. (2008). Screening of visible and UV radiation as a photoprotective mechanism in plants. Russ. J. Plant Physiol..

[24] Oliveira M.M., Daré R.G., Barizão É.O., Visentainer J.V., Romagnol M.B., Nakamura C.V., Truiti M.D.C.T. (2018). Photodamage attenuating potential of *Nectandra hihua* against UVB-induced oxidative stress in L292 fibroblasts. J. Photochem. Photobiol. B.

[25] Ribeiro F.M., Volpato H., Lazarin-Bidóia D., Desoti V.C., de Souza R.O., Fonseca M.J.V., Ueda-Nakamura T., Nakamura C.V., Silva S.O. (2018). The extended production of UV-induced reactive oxygen species in L929 fibroblast is attenuated by posttreatment with *Arrabidaea chica* through scavenging mechanisms. J. Photochem. Photobiol. B.

[26] Stockert J.C., Blázquez-Castro A., Cañate M., Horobin R.W., Villanueva A. (2012). MTT assay for cell viability: Intracellular localization of the formazan product is in lipid droplets. Acta Histochem..

[27] Estrada-Zúñiga M.E., Cruz-Sosa F., Rodríguez-Monroy M., Verde-Calvo J.R., Vernon-Carter E.J. (2009). Phenylpropanoid production in callus and cell suspension cultures of *Buddleja cordata* Kunth. Plant Cell Tissue Organ Cult..

[28] Khan S., Ullah H., Zhang L. (2019). Review: Bioactive constituents from *Buddleja* species. Pak. J. Pharm. Sci..

[29] Norman E.M. (2000). Flora Neotropica Monograph 81.

[30] Tatli I.I., Kahraman C., Akdemin Z.S., Ross R.W., Preedy V.R. (2015). Therapeutic activities of selected Scrophulariaceae and Buddlejaceae species and their secondary metabolites against neurodegenerative diseases. Bioactive Nutraceuticals and Dietary Supplements in Neurological and Brain Disease. Prevention and Therapy.

[31] Houghton P.J., Mensah A.Y., Lessa N., Hong L.Y. (2003). Terpenoids in *Buddleja*: Relevance to chemosystematics, chemical ecology and biological activity. Phytochemistry.

[32] García-Bores A.M., Hernández T., Arcienegas A.R., Benítez J.C., González M.R., López M., Vivar A.R., Avila J.G., Césped C.L., Sampierto D.A., Seigler D.L., Rai M. (2013). Photoprotective activity of some Mexican plant. Natural Antioxidants and Biocides from Wild Medicinal Plants.

[33] Ávila Acevedo J.G., Castañeda C.M., Benítez F.J., Durán D.A., Barroso V.R., Martínez C.G., Muñoz L.J., Martínez C.A., Romo de Vivar A. (2005). Photoprotective activity of *Buddleja scordioides*. Fitoterapia.

[34] Acevedo J.G.A., González A.M.E., Campos D.M.D.M., Flores J.D.C.B., Delgado T.H., Maya S.F., Contreras J.C., López J.L.M., Bores A.M.G. (2014). Photoprotection of *Buddleja cordata* extract against UVB-induced skin damage in SKH-1 hairless mice. BMC Complement. Altern. Med..

[35] Espinoza-González A.M., García-Bores A.M., Benítez-Flores J.C., Sandoval-Pérez E., González-Valle M.R.L., Céspedes C., Avila-Acevedo J.G. (2016). Photoprotective effect of verbascoside from *Buddleja cordata* in SKH-1 mice exposed to acute and chronic UV-B radiation. Boletín Latinoamericano y del Caribe de Plantas Medicinales y Aromáticas.

[36] Alipieva K., Korkina L., Orhan I.E., Georgiev M.I. (2014). Verbascoside-a review of its occurrence, (bio) synthesis and pharmacological significance. Biotechnol. Adv..

[37] Pesce M., Franceschelli S., Ferrone A., De LutIis M.A., Patruno A., Grilli A., Felaco M., Speranza L. (2015). Verbascoside down-regulates some pro-inflammatory signal transduction pathways by increasing the activity of tyrosine phosphatase, SHP-1 in the U937 cell line. J. Cell. Mol. Med..

[38] Han X., Wu Y.C., Meng M., Sun Q.S., Gao S.M., Sun H. (2018). Linarin prevents LPS-induced acute lung injury by suppressing oxidative stress and inflammation via inhibition of TXNIP/NLRP3 and NF-κB pathways. Int. J. Mol. Med..

[39] Estrada-Zúñiga M.E., Gutiérrez-Rebolledo G.A., Nieto-Trujillo A., Bernabé-Antonio A., Cruz Sosa F., Yanik T. (2019). *Buddleja* species distributed in Mexico against inflammatory diseases, their therapeutic activities, secondary metabolites and biotechnology. Recent Advances in Biological Research.

[40] Álvarez M.A., Álvarez M.A. (2014). Plant secondary metabolism. Plant Biotechnology for Health.

[41] Vázquez-Marquez A.M., Zepeda-Gómez C., Burrola-Aguilar C., Bernabé-Antonio A., Nieto-Trujillo A., Cruz-Sosa F., Rodríguez-Monroy M., Estrada-Zúñiga M.E. (2019). Effect of stirring speed on the production of phenolic secondary metabolites and growth of *Buddleja cordata* cells cultured in mechanically agitated bioreactor. Plant Cell Tissue Organ Cult.

[42] Arano-Varela H., Cruz-Sosa F., Estrada-Zúñiga M.E., Fernández F.J. (2020). Effects of phenylalanine and methyl jasmonate on verbascoside production in *Buddleja cordata* Kunth cell suspension cultures. S. Afr. J. Bot..

[43] Arano-Varela H., Fernández F.J., Estrada-Zúñiga M.E., Cruz-Sosa F. (2020). Verbascoside production in long-term *Buddleja cordata* Kunth cell suspension cultures. 3 Biotech.

[44] Gutiérrez-Rebolledo G.A., Estrada-Zúñiga M.E., Nieto-Trujillo A., Cruz-Sosa F., Jiménez-Arellanes M.A. (2018). In vivo anti-inflamatory activity and acute toxicity of methanolic extracts from wild plant leaves and cell suspension cultures of *Buddleja cordata* Kunth (Buddlejaceae). Rev. Mex. Ing. Quím..

[45] Gutiérrez-Rebolledo G.A., Estrada-Zúñiga M.E., Garduño-Siciliano L., García-Gutiérrez G.E., Reséndiz Mora C.A., Calderón-Amador J., Cruz-Sosa F. (2019). In vivo anti-arthritic effect and repeated dose toxicity of standardized methanolic extracts of *Buddleja cordata* Kunth (Scrophulariaceae) wild plant leaves and cell culture. J. Ethnopharmacol..

[46] Phol J., Christophers E. (1979). Growth characteristics of skin fibroblast and 3T3 cells entrapped by polymerizing fibrin. In Vitro.

[47] Menon G.K., Pappas A. (2015). Chapter 2 Skin basics; structure and function. Lipids and Skin Health.

[48] Zeng Q., Zhou F., Lei L., Chen J., Lu J., Zhou J., Cao K., Xia F., Ding S., Huang L. (2017). *Ganodena lucidum* polysaccharides protect fibroblast against UVB-induce photoaging. Mol. Med. Rep..

[49] Chung Y.H., Jeong S.A., Choi H.S., Ro S., Lee J.S., Park J.K. (2018). Protective effects of ginsenoside Rg2 and astaxanthin mixture against UVB-induce DNA damage. Anim. Cells Syst..

[50] Liu W., Otkur W., Zhang Y., Li Q., Ye Y., Zang L., He H., Hayashi T., Tashiro S., Onodera S. (2013). Silibinin protects murine fibroblast L929 cells from UVB-induced apoptosis through the simultaneous inhibition of ATM-p53 pathway and autophagy. FEBS J..

[51] Petrucci R., Astolfi P., Greci L., Firuzi O., Saso L., Marrosu G. (2007). A spectroelectrochemical and chemical study on oxidation of hydroxycinnamic acids in aprotic medium. Electrochim. Acta..

[52] Narayanan D.L., Saladin R.N., Fox J.L. (2010). Ultraviolet radiation and skin cancer. Int. J. Dermatol..

[53] Khare S., Singh N.B., Singh A., Hussain I., Niharika K., Yadav V., Bano C., Yadav R.K., Amist N. (2020). Plant secondary metabolites synthesis and their regulations under biotic and abiotic constraints. J. Plant Biol..

[54] Cardinali A., Pati S., Minervini F., D’Antuono I., Linsalata V., Lattanzio V. (2012). Verbascoside, isoverbascoside, and their derivatives recovered from olive mill wastewater as possible food antioxidants. J. Agric. Food Chem..

[55] Li Y., Gan L., Li G.Q., Deng L., Zhang X., Deng Y. (2014). Pharmacokinetics of plantamajoside and acteoside from *Plantago asiatica* in rats by liquid chromatography-mass spectrometry. J. Pharm. Biomed. Anal..

[56] Fraga C.G., Galleano M., Verstraeten S.V., Oteiza P.I. (2010). Basic biochemical mechanisms behind the health benefits of polyphenols. Mol. Aspects. Med..

[57] Ried A.M., Juvonen R., Huuskonen P., Lehtonen M., Pasanen M., Lall M. (2019). In vitro human metabolism and inhibitory potency of verbascoside for CYP enzymes. Molecules.

[58] Kapepula P.M., Mbombo Munditshi P., Frank T., Mouithys-Mickalad A., Mumba Ngoyi D., Kalenda P.D.T., Kabamba Ngombe N., Serteyn D., Tits M., Frédérich M. (2017). Antioxidant potentiality of three herbal teas consumed in Bandundu rural areas of Congo. Nat. Prod. Res..

[59] Zhao C., Dodin G., Yuan C., Chen H., Zheng R., Jia Z., Fan B.T. (2005). “In vitro” protection of DNA from fenton reaction by plant polyphenol verbascoside. Biochim. Biophy. Acta.

[60] Rui-Chuan C., Jin-Hua S., Shan-Min Y., Ji L., Tian-Jiao W., Hong Z. (2002). Effect of isoverbascoside, a phenylpropanoid glycoside antioxidant, on proliferation and differentiation of human gastric cancer cell. Acta Pharmacol. Sin..

[61] Crivellari I., Vertuani S., Lim Y., Cervellati F., Baldisserotto A., Mandredini S., Valacchi G. (2018). ES2 as a novel verbascoside-derived compound in the treatment of cutaneous wound healing. Cosmetics.

[62] Henn J.G., Steffens L., de Moura Sperotto N.D., de Souza Ponce B., Veríssimo R.M., Boaretto F.B.M., Hassemer G., Péres V.F., Schirme H., Picada J.N. (2019). Toxicological evaluation of a standardized hydroethanolic extract from leaves of *Plantago australis* and its major compound, verbascoside. J. Ethnopharmacol..

[63] Kawakami T., Xiao W., Yasudo H., Kawakami Y. (2012). Regulation of proliferation, survival, differentiation, and activation by the signaling platform for SHP-1 phosphatase. Adv. Enzym. Regul..

[64] Poole A.W., Jones M.L. (2005). A SHPing tale: Perspectives on the regulation of SHP-1 and SHP-2 tyrosine phosphatases by the C-terminal tail. Cell Signal.

[65] Kostyuk V., Potapovich A., Suhan T., De Luca C., Pressi G., Dal Toso R., Korkina L. (2008). Plant polyphenols against UV-C-induced cellular death. Planta Med..

[66] Pastore S., Potapovich A., Kostyuk V., Mariani V., Lulli D., De Luca C., Korkina L. (2009). Plant polyphenols effectively protect HaCaT cells from ultraviolet C-Triggered necrosis and suppress inflammatory chemokine expression. Ann. N. Y. Acad. Sci..

[67] Speranza L., Fransceschelli S., Pesce M., Reale M., Menghini L., Vinciguerra I., De LuitiIss M.A., Felaco M., Grilli A. (2010). Antiinflamatory effects in THP-1 cells treated with verbascoside. Phytother. Res..

[68] De Moura Sperotto N.D., Steffens L., Veríssimo R.M., Henn J.G., Péres V.F., Vianna P., Chies J.A.B., Roehe A., Saffi J., Moura D.M. (2018). Wound healing and anti-inflammatory activities induced by *Plantango australis* hydroethanolic extract standardized in verbascoside. J. Ethnopharmacol..

[69] Liu M.J., Li J.X., Guo H.Z., Lee K.M., Qin L., Chan K.M. (2003). The effects of verbascoside on plasma lipid peroxidation level and erythrocyte membrane fluidity immobilization in rabbits: A time course study. Life Sci..

[70] Singh M., Devi S., Rana V.S., Kumar J., Ahluwalia V. (2019). Delivery of phytochemicals by liposome cargos: Recent progress, challenges and opportunities. J. Microencapsul..

[71] Rodrigo R., Miranda A., Vergara L. (2011). Modulation of endogenous antioxidant system by wine polyphenols in huma disease. Clin.Chim. Acta.

[72] Zhou F., Xu T., Zhao Y., Song H., Zhang L., Wu X., Lu B. (2018). Chitosan-coated liposomes as delivery systems for improving the stability and oral bioavailability of acteoside. Food Hydrocol..

[73] Isacchi B., Bergonzi M.C., Iacopi R., Ghelardini C., Galeotti N., Bilia A.R. (2017). Liposomal formulation to increase stability and prolong antineuropathic activity of verbascoside. Planta Med..

[74] Sinico C., Caddeo C., Valenti D., Fadda A.M., Bila A.R., Vincieri F.F. (2008). Liposomes as carriers for verbascoside: Stability and skin permeation studies. J. Liposome Res..

[75] Ambrosone L., Guerra G., Cinelli M., Filippelli M., Mosca M., Vizzarri F., Giorgio D., Costagliola C. (2014). Corneal epitelial wound healing promoted by verbascoside-based liposomal eyedrops. BioMed Res. Int..

[76] Schuch A.P., Moreno N.C., Schuch N.J., Menk C.F.M., Garcia C.C.M. (2017). Sunlight damage to cellular DNA: Focus on oxidatively generated lesions. Free Radic. Biol. Med..

[77] Chen A.C., Halliday G.M., Damian D.L. (2013). Non-melanoma skin cancer: Carcinogenesis and chemoprevention. Pathology.

[78] Canul M.E.A., Rocher C.C., Zapata H.R., Trujillo H.P. (2015). Cáncer de piel en Yucatán: Un estudio epidemiológico de 10 años. Dermatología Cosmética Médica Quirúrgica.

[79] Pinedo-Vega J.L., Castañeda-López R., Dávila-Rangel J.I., Mireles-García F., Ríos-Martínez C., López Saucedo A. (2014). Incidencia de cáncer de piel en Zacatecas. Rev. Med. Inst. Mex. Seguro Soc..

[80] Alfaro-Sánchez A., García-Hidalgo L., Casados-Vergara R., Rodríguez-Cabral R., Piña-Osuna A.K., Sánchez-Ramos A. (2016). Cáncer de piel. Epidemiología y variedades histológicas, estudio de cinco años en el noreste de México. Dermatol. Rev. Mex..

[81] Antonio-Gutiérrez O.T., López Mano A., Paulo E., Ramírez-Corona N. (2015). Métodos para la determinación de la dosis de radiación ultravioleta de onda corta (UVC) en alimentos. TSIA.

