# Supplementary material for: Photoprotective Activity of Buddleja cordata Cell Culture Methanolic Extract on UVB-irradiated 3T3-Swiss Albino Fibroblasts
Source: Plants (Basel). 2021 Jan 30;10(2):266. doi: 10.3390/plants10020266 (PMC7912278; doi:10.3390/plants10020266)
Supplement: Supplementary file 1 [file plants-10-00266-s001.pdf]

plants

MDPI

Article

Photoprotective Activity of *Buddleja cordata* Cell Culture Methanolic Extract on UVB-Irradiated 3T3-Swiss Albino Fibroblasts

Milton Abraham Gómez-Hernández [1], Miriam Verónica Flores-Merino [2,*], Jesús Enrique Sánchez-Flores [2], Cristina Burrola-Aguilar [3], Carmen Zepeda-Gómez[4], Aurelio Nieto-Trujillo [3] and María Elena Estrada-Zúñiga [3,*]

(**a**)

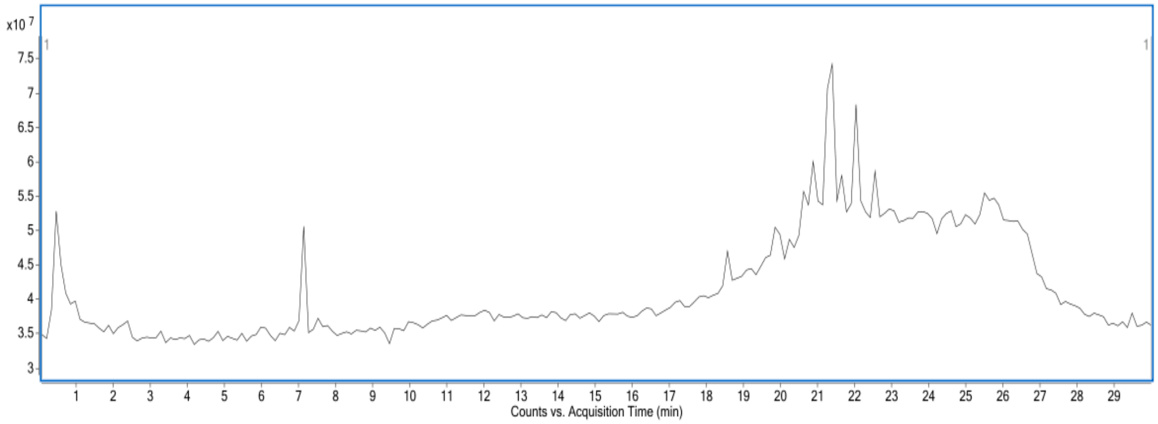

(**b**)

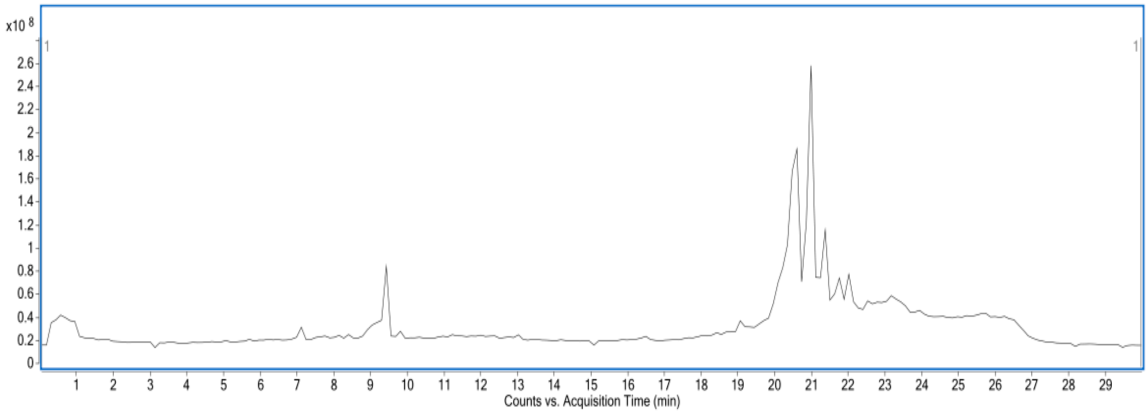

Figure S1. LC/MS chromatogram in electrospray (**a**) negative-ion and (**b**) positive-ion mode of the methanolic extract of *Buddleja cordata* cell culture, where 2-(3-hydroxyphenyl)ethanol 1′-glucoside, verbascoside, isoverbascoside, beta-sitosterol 3-O-beta-D-galactopyranoside, and 3-hydroxystigmast-5-en-7-one were detected at 1.959, 7.162, 7.177, 24.439, and 26.261 min, respectively..

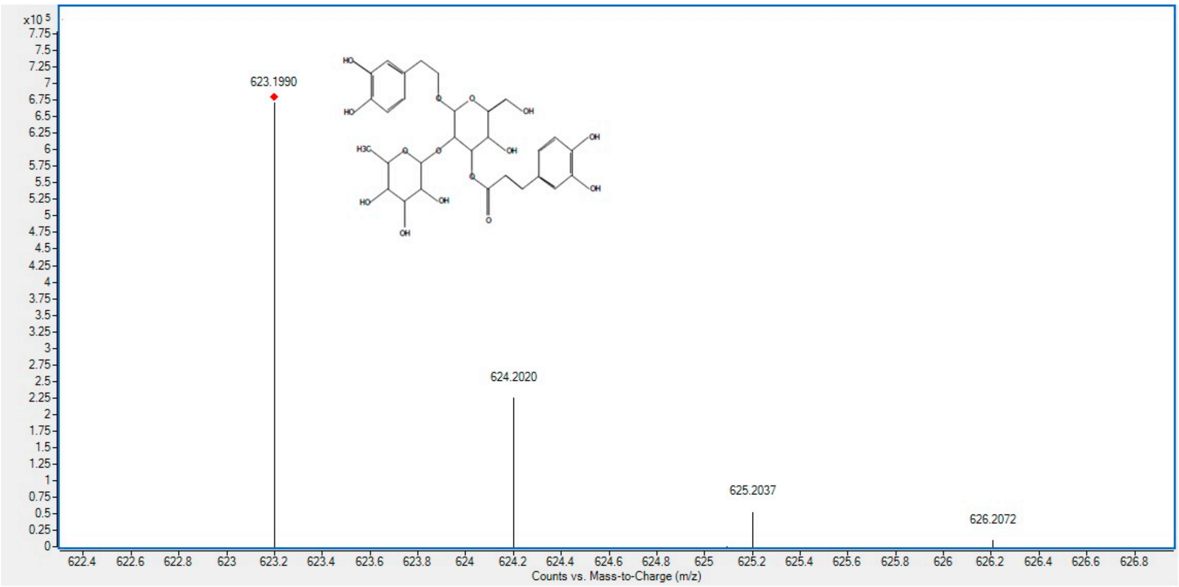

Peak List

m/z	z	Abund	Name	Formula	Ion	Score (DB)	Hits (DB)
623.1988	1	471312.53					
659.1753	1	483234.09	Magnoloside A	C29 H36 O15	(M+Cl)-	59.2	5
660.178	1	155330.06		C29 H36 O15	(M+Cl)-		

Figure S2. Mass spectrum of verbascoside ($C_{29}H_{36}O_{15}$) in electrospray negative-ion mode detected in the methanolic extract of *Buddleja cordata* cell culture.

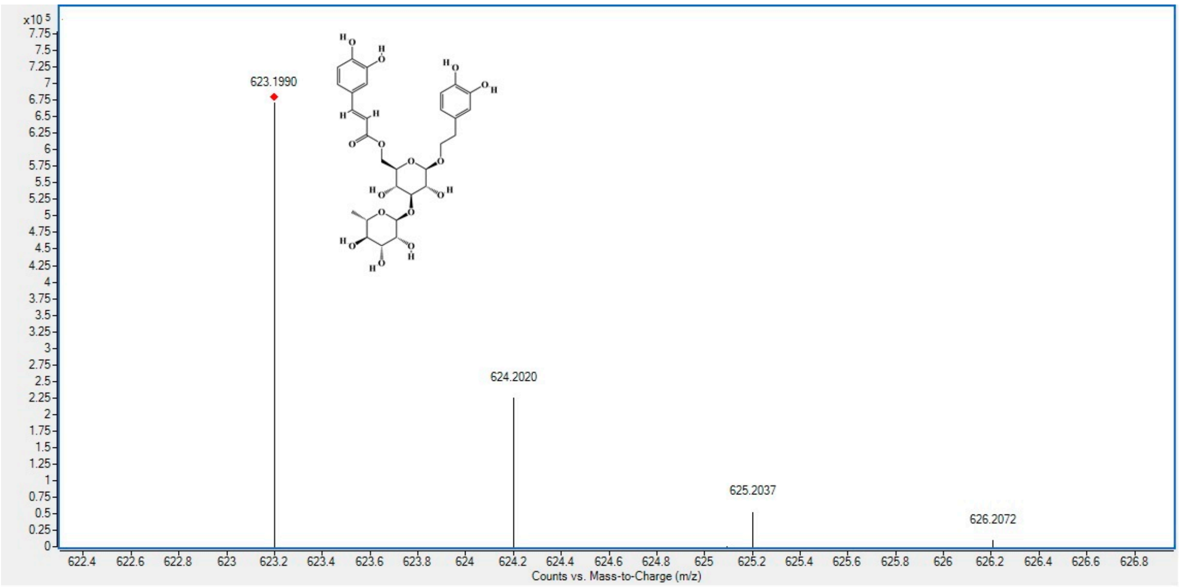

Peak List

m/z	z	Abund	Name	Formula	Ion	Score (DB)	Hits (DB)
623.1991	1	779153.94	Isoacteoside	C29 H36 O15	(M-H)-	98.74	10
624.2019	1	245760.28		C29 H36 O15	(M-H)-		
625.2037	1	59310.46		C29 H36 O15	(M-H)-		
626.2061	1	10129.47		C29 H36 O15	(M-H)-		

Figure S3. Mass spectrum of isoverbascoside ($C_{29}H_{36}O_{15}$) in electrospray negative-ion mode detected in the methanolic extract of *Buddleja cordata* cell culture.

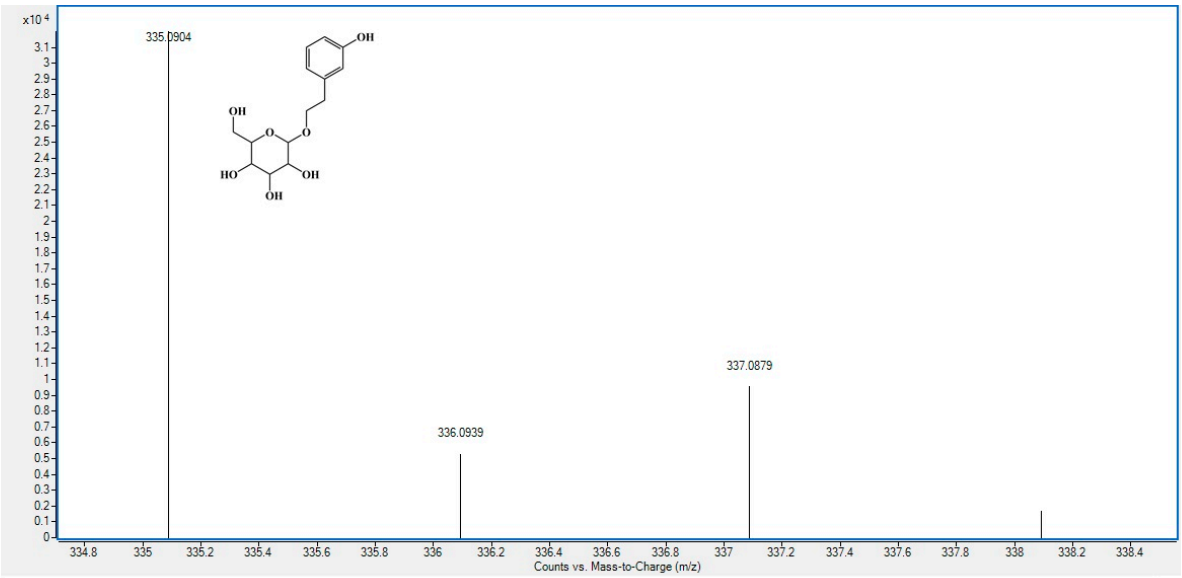

Peak List

m/z	z	Abund	Name	Formula	Ion	Score (DB)	Hits (DB)
335.0903	1	148689.48	2-(3-Hydroxyphenyl)ethanol 1'-glucoside	C14 H20 O7	(M+Cl)-	98.58	3
336.0933	1	22026.62		C14 H20 O7	(M+Cl)-		
337.088	1	46244.76		C14 H20 O7	(M+Cl)-		
338.0912	1	6857.5		C14 H20 O7	(M+Cl)-		

Figure S4. Mass spectrum of 2-(3-Hydroxyphenyl)ethanol 1'-glucoside ($C_{14}H_{20}O_7$) in electrospray negative-ion mode detected in the methanolic extract of *Buddleja cordata* cell culture.

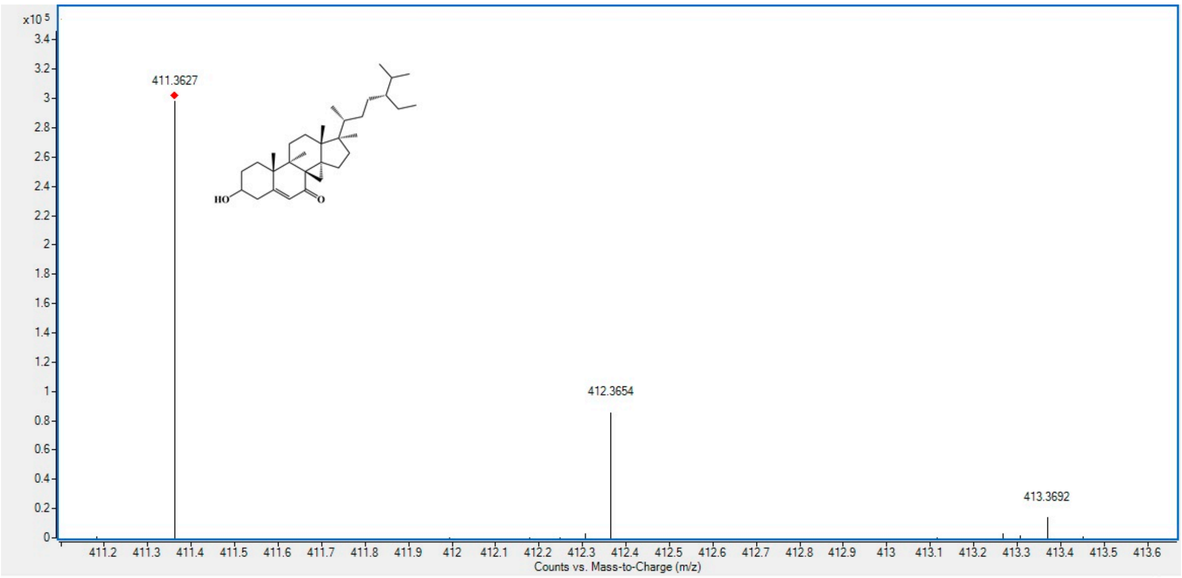

Peak List

m/z	z	Abund	Name	Formula	Ion	Score (DB)	Hits (DB)
411.3625	1	250862.03	3-Hydroxystigmast-5-en-7-one	C29 H48 O2	(M+H)+[-H2O]	99.31	10
412.3654	1	77257.83		C29 H48 O2	(M+H)+[-H2O]		
413.3689	1	11234.64		C29 H48 O2	(M+H)+[-H2O]		

Figure S5. Mass spectrum of 3-hydroxystigmast-5-en-7-one ($C_{29}H_{48}O_2$) in electrospray positive-ion mode detected in the methanolic extract of *Buddleja cordata* cell culture.

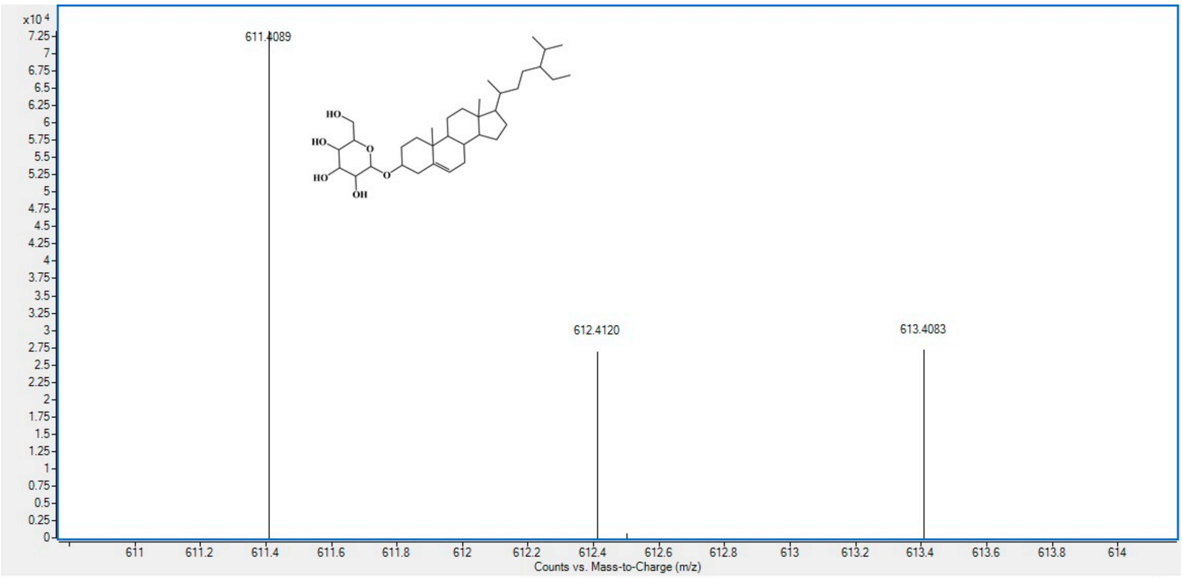

Peak List

m/z	z	Abund	Name	Formula	Ion	Score (DB)	Hits (DB)
611.4084	1	192000.34	beta-Sitosterol 3-O-beta-D-galactopyranoside	C35 H60 O6	(M+Cl)-	81.99	10
612.4114	1	72527.77		C35 H60 O6	(M+Cl)-		
613.4073	1	71121		C35 H60 O6	(M+Cl)-		

Figure S6. Mass spectrum of beta-Sitosterol 3-O-beta-D-galactopyranoside ($C_{35}H_{60}O_6$) in electrospray negative-ion mode detected in the methanolic extract of *Buddleja cordata* cell culture.

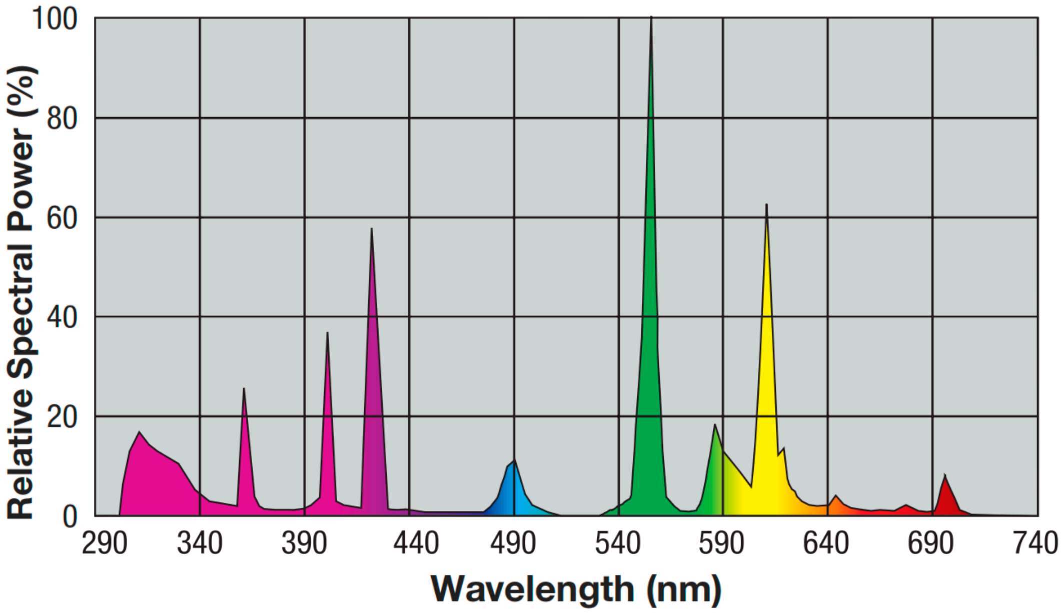

Figure S7. UV–visible spectrum of UVB150 Exo Terra Reptile 26W lamp used for UVB irradiation of 3T3-Swiss albino fibroblasts. The UVB150 Exo Terra Reptile has a high UVB output similar to that of sunlight in deserts (http://www.exo-terra.com/en/products/reptile_uvb150.php)
